# Storywrangler: A massive exploratorium for sociolinguistic, cultural, socioeconomic, and political timelines using Twitter

**DOI:** 10.1126/sciadv.abe6534

**Published:** 2021-07-16

**Authors:** Thayer Alshaabi, Jane L. Adams, Michael V. Arnold, Joshua R. Minot, David R. Dewhurst, Andrew J. Reagan, Christopher M. Danforth, Peter Sheridan Dodds

**Affiliations:** 1 Vermont Complex Systems Center, University of Vermont, Burlington, VT 05405, USA.; 2 Computational Story Lab, University of Vermont, Burlington, VT 05405, USA.; 3 Department of Computer Science, University of Vermont, Burlington, VT 05405, USA.; 4 Charles River Analytics, Cambridge, MA 02138, USA.; 5 MassMutual Data Science, Boston, MA 02110, USA.

## Abstract

In real time, Twitter strongly imprints world events, popular culture, and the day-to-day, recording an ever-growing compendium of language change. Vitally, and absent from many standard corpora such as books and news archives, Twitter also encodes popularity and spreading through retweets. Here, we describe Storywrangler, an ongoing curation of over 100 billion tweets containing 1 trillion 1-grams from 2008 to 2021. For each day, we break tweets into 1-, 2-, and 3-grams across 100+ languages, generating frequencies for words, hashtags, handles, numerals, symbols, and emojis. We make the dataset available through an interactive time series viewer and as downloadable time series and daily distributions. Although Storywrangler leverages Twitter data, our method of tracking dynamic changes in *n*-grams can be extended to any temporally evolving corpus. Illustrating the instrument’s potential, we present example use cases including social amplification, the sociotechnical dynamics of famous individuals, box office success, and social unrest.

## Teaser

We present Storywrangler, an interactive cultural exploratorium of phrase popularity using 100 billion tweets in 100 languages.

## INTRODUCTION

Our collective memory lies in our recordings—in our written texts, artworks, photographs, audio, and video—and in our retellings and reinterpretations of that which becomes history. The relatively recent digitization of historical texts, from books (*1*–*4*) to news (*5*–*8*) to folklore (*9*–*12*) to governmental records (*13*), has enabled compelling computational analyses across many fields (*10*, *14*, *15*), but books, news, and other formal records only constitute a specific type of text—carefully edited to deliver a deliberate message to a target audience. Large-scale constructions of historical corpora also often fail to encode a fundamental characteristic: popularity (i.e., social amplification). How many people have read a text? How many have retold a news story to others?

For text-based corpora, we are confronted with the challenge of sorting through different aspects of popularity of *n*-grams—sequences of *n* “words” in a text that are formed by contiguous characters, numerals, symbols, emojis, etc. An *n*-gram may or may not be part of a text’s lexicon, as the vocabulary of a text gives a base sense of what that text may span meaningwise (*16*). For texts, it is well established that *n*-gram frequency-of-usage (or Zipf) distributions are heavy tailed (*17*). Problematically, this essential character of natural language is readily misinterpreted as indicating cultural popularity. For a prominent example, the Google Books *n*-gram corpus (*1*), which, in part, provides inspiration for our work here, presents year-scale, *n*-gram frequency time series where each book, in principle, counts only once (*2*). All cultural fame is stripped away. The words of George Orwell’s *1984* or Rick Riordan’s *Percy Jackson* books, indisputably read and reread by many people around the world, count as equal to the words in the least read books published in the same years. However, time series provided by the Google Books *n*-gram viewer have regularly been erroneously conflated with the changing interests of readers [e.g., the apparent decline of sacred words (*2*, *18*–*21*)]. Further compounded with an increase of scientific literature throughout the 20th century, the corpus remains a deeply problematic database for investigations of sociolinguistic and cultural trends. It is also very difficult to measure cultural popularity. For a given book, we would want to know sales of the book over time, how many times the book has been actually read, and to what degree a book becomes part of broader culture. Large-scale corpora capturing various aspects of popularity exist (*15*) but are hard to compile, as the relevant data are either prohibitively expensive or closed (e.g., Facebook) and, even when accessible, may not be consistently recorded over time (e.g., Billboard’s Hot 100).

Now, well into the age of the internet, our recordings are vast, inherently digital, and capable of being created and shared in the moment. People, news media, governmental bodies, corporations, bots, and many other entities all contribute constantly to giant social media platforms. When open, these services provide an opportunity for us to attempt to track myriad statements, reactions, and stories of large populations in real time. Social media data allow us to explore day-to-day conversations by millions of ordinary people and celebrities at a scale that is scarcely conventionalized and recorded. Crucially, when sharing and commenting mechanisms are native to a social media platform, we can quantify popularity of a trending topic and social amplification of a contemporary cultural phenomenon.

Here, we present Storywrangler, a natural language processing framework that extracts, ranks, and organizes *n*-gram time series for social media. Storywrangler provides an analytical lens to examine discourse on social media, carrying both the voices of famous individuals—political figures and celebrities—and the expressions of the many. With a complex, ever-expanding fabric of time-stamped messages, Storywrangler allows us to capture storylines in over 150 languages in real time.

For a primary social media source, we use Twitter for several reasons, while acknowledging its limitations. Our method of extracting and tracking dynamic changes of *n*-grams can, in principle, be extended to any social media platform (e.g., Facebook, Reddit, Instagram, Parler, 4Chan, and Weibo).

Twitter acts as a distributed sociotechnical sensor system (*22*, *23*). Using Storywrangler, we can trace major news events and stories, from serious matters such as natural disasters (*24*–*28*) and political events (*29*) to entertainment such as sports, music, and movies. Storywrangler also gives us insights into discourse around these topics and myriad others including, violence, racism, inequality, employment, pop culture (e.g., fandom), fashion trends, health, metaphors, emerging memes, and the quotidian.

We can track and explore discussions surrounding political and cultural movements that are born and nurtured in real time over social media with profound ramifications for society (e.g., #MeToo, #BlackLivesMatter, and #QAnon). Modern social movements of all kinds may develop a strong imprint on social media, over years in some cases, before becoming widely known and discussed.

Twitter and social media, in general, differ profoundly from traditional news and print media in various dimensions. Although amplification is deeply uneven, vast numbers of people may now express themselves to a global audience on any subject that they choose (within limits of a service and not without potential consequences). Unlike journalists, columnists, or book authors, people can instantly record and share messages in milliseconds. From a measurement perspective, this is a far finer temporal resolution than would be reasonably needed to explore sociocultural phenomena or reconstruct major events. The eye witness base for major events is now no longer limited to those physically present because of growing, decentralized livestreaming through various social media platforms. Social media thus enables, and not without peril, a kind of mass distributed journalism.

A crucial feature of Storywrangler is the explicit encoding of *n*-gram popularity, which is enabled by Twiter’s social amplification mechanisms: retweets and quote tweets. For each day and across languages, we create Zipf distributions for the following: (i) *n*-grams from originally authored messages (OT), excluding all retweeted material (RT), and (ii) *n*-grams from all Twitter messages (AT). For each day, we then have three key levels of popularity: *n*-gram lexicon, *n*-gram usage in organic tweets (originally authored tweets), and the rate at which a given *n*-gram is socially amplified (i.e., retweeted) on the platform. Our data curation using Storywrangler yields a rich dataset, providing an interdisciplinarity resource for researchers to explore transitions in social amplification by reconstructing *n*-gram Zipf distributions with a tunable fraction of retweets.

We structure our paper as follows. In Materials and Methods, we briefly describe our instrument, dataset, and the Storywrangler site, which provides day-scale *n*-gram time series datasets for *n* = 1, 2, and 3, both as time series and as daily Zipf distributions. In Results, we showcase a group of example analyses, arranged by increasing complication: simple *n*-gram rank time series (see the “Basic rank time series” section); qualitative comparison to other prominent social signals of Google Trends and cable news (see the “Comparison to other signals” section); contagiograms, time series showing social amplification (see the “Contagiograms” section); analysis for identifying and exploring narratively trending storylines (see the “Narratively trending storylines” section); and an example set of case studies bridging *n*-gram time series with disparate data sources to study famous individuals, box office success, and social unrest (see the “Case studies” section). In our concluding remarks in Discussion, we outline some potential future developments for Storywrangler.

## RESULTS

### Basic rank time series

**Fig. 1:**
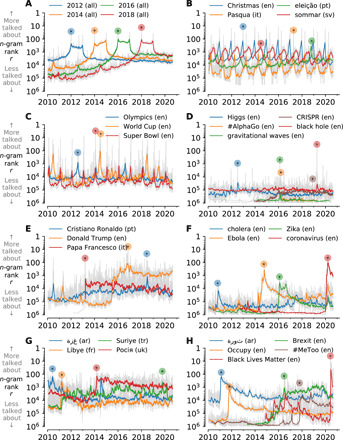
Thematically connected *n*-gram time series. For each *n*-gram, we display daily rank in gray overlaid by a centered monthly rolling average (colored lines) and highlight the *n*-gram’s overall highest rank with a solid disk. (**A**) Anticipation and memory of calendar years for all of Twitter. (**B**) Annual and periodic events: Christmas in English (blue), Easter in Italian (orange), election in Portuguese (green), and summer in Swedish (red). (**C**) Attention around international sports in English: Olympics (blue), FIFA World Cup (orange), and Super Bowl (red). (**D**) Major scientific discoveries and technological innovations in English. (**E**) Three famous individuals in relevant languages: Ronaldo (Portuguese), Trump (English), and Pope Francis (Italian). (**F**) Major infectious disease outbreaks. (**G**) Conflicts: Gaza in Arabic (blue), Libya in French (orange), Syria in Turkish (green), and Russia in Ukrainian (red). (**H**) Protest and movements: Arab Spring (Arabic word for “revolution,” blue), Occupy movement (English, orange), Brexit campaign (English, green), #MeToo movement (English, brown), and Black Lives Matter protests (English, red).

In Fig. 1, we show rank time series for eight sets of *n*-grams from all tweets (i.e., including retweets). The *n*-gram groups move from simple to increasingly complex in theme, span a number of languages, and display a wide range of sociotechnical dynamics. Because of an approximate obeyance of Zipf’s law (*f* ∼ *r*^−θ^), we observe that normalized frequency of usage time series matches rank time series in basic form. We use rank as the default view for its straightforwardness.

Starting with time and calendars, Fig. 1A gives a sense of how years are mentioned on Twitter. The dynamics show an anticipatory growth, plateau, and then rapid decay, with each year’s start and finish marked by a spike.

Figure 1 (B and C) shows calendrically anchored rank time series for seasonal, religious, political, and sporting events that recur at the scale of years in various languages. Periodic signatures at the day, week, and year scale are prominent on Twitter, reflecting the dynamics of the Earth, Moon, and Sun. Easter (shown in Italian), in particular, combines cycles of all three. Major sporting events produce time series with strong anticipation and can reach great heights of attention as exemplified by a peak rank of *r* = 3 for “Super Bowl” on 2 February 2014.

We move to scientific announcements in Fig. 1D with the 2012 discovery of the Higgs boson particle (blue), detection of gravitational waves (green), and the first imaging of a black hole (red). For innovations, we show the time series of “#AlphaGo,” the first artificial intelligence program to beat the human Go champion (orange), along with the development of CRISPR technology for editing genomes (brown). We see that time series for scientific advances generally show shock-like responses with little anticipation or memory (*30*). CRISPR is an exception for these few examples as through 2015, it moves to a higher, enduring state of being referenced.

Fame is the state of being talked about, and famous individuals are well reflected on Twitter (*31*). In Fig. 1E, we show time series for the Portuguese football player Cristiano Ronaldo, the 45th U.S. president Donald Trump, and Pope Francis (Papa Francesco in Italian). All three show enduring fame, following sudden rises for both Trump and Pope Francis. On 9 November 2016, the day after the U.S. election, “Donald Trump” rose to rank *r* = 6 among all English 2-grams.

In Fig. 1F, we show example major infectious disease outbreaks over the past decade. Time series for pandemics are shocks followed by long relaxations, resurging both when the disease returns in prevalance and also in the context of new pandemics. Cholera, Ebola, and Zika all experienced elevated discussion within the context of the COVID-19 pandemic.

In Fig. 1G, we show *n*-gram signals of regional unrest and fighting. The word for Gaza in Arabic tracks events of the ongoing Israeli-Palestinian conflict. The time series for “Libye” points to Opération Harmattan, the 2011 French and North Atlantic Treaty Organization military intervention in Libya. Similarly, the time series for “Syria” in Turkish indicates the dynamics of the ongoing Syrian civil war on the region, and the buildup and intervention of the Russian military in Ukraine are mirrored by the use of the Ukrainian word for “Russia.”

In Fig. 1H, we highlight protests and movements. Both the time series for “revolution” in Arabic and “Occupy” in English show strong shocks followed by slow relaxations over the following years. The social justice movements represented by “#MeToo” and “Black Lives Matter” appear abruptly, and their time series show slow decays punctuated by shocks returning them to higher ranks. Black Lives Matter resurged after the murder of George Floyd, with the highest 1-day rank of *r* = 4 occurring on 2 June 2020. By contrast, the time series of “Brexit,” the portmanteau for the movement to withdraw the United Kingdom from the European Union, builds from around the start of 2015 to the referendum in 2016, and then continues to climb during the years of complicated negotiations to follow.

### Comparison to other signals

**Fig. 2:**
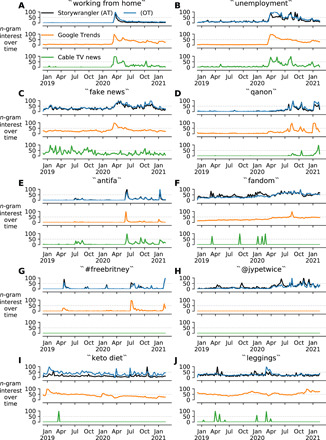
Comparison between Twitter, Google Trends, and cable news. All time series are rescaled between 0 (low interest) and 100 (peak interest) to represent rate of usage relative to the highest point for the given time window. For each *n*-gram (case insensitive), we display weekly interest over time using Storywrangler for all tweets (AT, black) and originally authored tweets (OT, blue), comparing that with Google Trends (orange) (*32*) and cable TV news (green) (*8*). (**A**) Similar social attention to working from home across media sources amid the COVID-19 pandemic. (**B**) Discourse of unemployment continues to fluctuate on Twitter in contrast to other mainstream media sources. (**C**) Usage of the bigram fake news. (**D**) Discussion of the QAnon conspiracy theory. (**E**) Mentions of “Antifa” on digital media. (**F**) Social attention of fans in various arenas as part of the ever-changing pop culture. (**G**) A growing social movement organized by longtime fans of Britney Spears regarding her conservatorship. (**H**) Official Twitter handle for the South Korean K-pop band: Twice. (**I**) Conversations surrounding fitness trends, which occasionally pop up in news via commercial advertisement. (**J**) Volatility of collective attention to fashion trends.

To highlight key differences that Storywrangler offers in contrast to other data sources, we display a few example comparisons in Fig. 2. In particular, we compare the usage rate for a set of *n*-grams using Storywrangler, Google Trends (*32*), and the Stanford cable TV news analyzer (*8*).

Each data source has its own unique collection scheme that is most appropriate to that venue. Google Trends provides search interest scaled relative to a given region and time. While Storywrangler is based on daily *n*-gram Zipf distributions, the Stanford cable TV news analyzer collects transcripts from most cable news outlets and breaks them into *n*-grams, recording screen time (seconds per day) for each term (*8*).

For the purpose of comparing *n*-gram usage across several disparate data sources, we take the weekly rate of usage for each term (case insensitive) and normalize each time series between 0 and 100 relative to the highest observed point within the given time window. A score of 100 represents the highest observed interest in the given term over time, while a value of 0 reflects low interest of that term and/or insufficient data. We display weekly interest over time for a set of 10 terms using Storywrangler for all tweets (AT, black) and originally authored tweets (OT, blue), Google trends (orange), and cable news (green).

In Fig. 2A, we show how usage of the trigram “working from home” peaks during March 2020 amid the COVID-19 pandemic. Although the term may be used in different contexts in each respective media source, we observe similar attention signals across all three data sources.

Similarly, Fig. 2B reveals increased mentions of “unemployment” on all media platforms during the U.S. national lockdown in April 2020. Individuals searching for unemployment claim forms could be responsible for the Google Trends spike, while news and social media usage of the term resulted from coverage of the economic crisis induced by the pandemic. The time series for unemployment continues to fluctuate on Twitter, with distinct patterns across all tweets and originally authored tweets.

In Fig. 2C, we see the bigram “fake news” roiling across social media and news outlets, reflecting the state of political discourse in 2020. This period saw the most sustained usage of the term since its initial spike following the 2016 U.S. election. The term was prominently searched for on Google in March 2020 during the early stages of the Coronavirus pandemic, but no corresponding spike is seen in cable news.

In Fig. 2D, the time series reveal attention to the “QAnon” conspiracy theory on social media and Google Trends starting in mid-2020. Using Storywrangler, we note a spike of “qanon” following Trump’s remarks regarding violent far-right groups during the first presidential debate on 29 September 2020. We see another spike of interest in October 2020 in response to the news about a kidnapping plot of the governor of Michigan by extremists. Although the time series using both Storywrangler and Google Trends show sustained usage of the term in 2020, news outlets do not exhibit similar patterns until the U.S. Capitol insurrection on 6 January 2021.

Figure 2E shows mentions of “antifa,” a political movement that drew attention in response to police violence during protests of the murder of George Floyd. We note that mentions surged again in response to false flag allegations in the wake of the Capitol attack, most prominently on Twitter.

In Fig. 2F, we display interest over time of the term “fandom,” a unigram that is widely used to refer to a group of people that share a common interest in creative genres, celebrities, fashion trends, modern tech, hobbies, etc. While this cultural phenomenon is rarely ever recorded by traditional news outlets, it dates back to the enormous fan base of Sherlock Holmes as one of the earliest signs of modern fandom, with public campaigners mourning the figurative death of their fictional character in 1893 (*33*). This cultural characteristic cannot be easily captured with data sources such as Google Books or search data. Nonetheless, it is intrinsic to nonmainstream media, illustrating the collective social attention of fans in various arenas as part of the ever-changing digital pop culture.

Figure 2G shows a recent example where longtime fans of the pop music star, Britney Spears, organized and launched a social media support campaign in light of the controversy surrounding her conservatorship. Although the movement dates back to 2009, we see a surge of usage of the hashtag “#FreeBritney” in July 2020, after an interview with Britney’s brother, revealing some personal details about her struggles and reigniting the movement on social media. The social movement has recently gained stronger cultural currency after the release of a documentary film by the New York Times in 2021.

Moreover, Fig. 2H shows interest over time of a popular South Korean pop band, “Twice.” Although the official handle of the band on Twitter (“jypetwice”) is virtually absent in other data sources, fans and followers use handles and hashtags regularly on Twitter to promote and share their comments for their musical bands.

In Fig. 2 (I and J), we see how communications and marketing campaigns of fitness trends such as “keto diet” and fashion trends such as leggings and athleisure receive sustained interest on Twitter while only occasionally popping up in news via commercial advertisements on some cable channels.

### Contagiograms

While rank time series for *n*-grams give us the bare temporal threads that make up the tapestries of major stories, our dataset offers more dimensions to explore. Per our introductory remarks on the limitations of text corpora, the most important enablement of our database is the ability to explore story amplification.

**Fig. 3:**
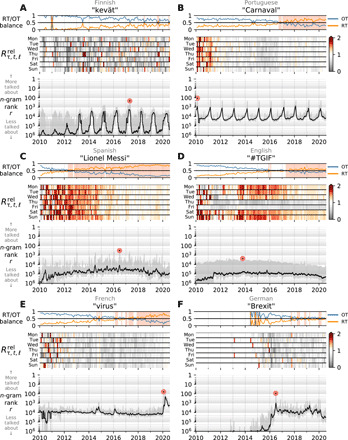
Contagiograms: Augmented time series charting the social amplification of *n*-grams. In each contagiogram, above the basic *n*-gram rank time series, the top panel displays the monthly relative usage of each *n*-gram, *R*_τ, *t*, 𝓁_ (Eq. 1), indicating whether they appear organically in new tweets (OT, blue) or in retweeted content (RT, orange). The shaded areas denote months when the balance favors spreading, suggestive of story contagion. The middle (second) panel then shows retweet usage of an *n*-gram relative to the background rate of retweeting, $R_{\tau ,t,\ell }^{\text{rel}}$ (Eq. 2). (**A** and **B**) The seasonal cycle of the 1-gram spring in Finnish is different from the annual cycle of the word Carnaval in Portuguese. Spring is often mentioned in organic tweets, while the balance of the word Carnaval favors retweets exceeding the social contagion threshold starting from 2017. (**C**) The time series for “Lionel Messi” in Spanish tweets exhibits a similar pattern of social amplification as a famous soccer player who is talked about regularly. (**D**) The hashtag #TGIF (“Thank God It’s Friday”) shows a strong weekly cycle, relatively unamplified on Thursday and Friday. (**E**) The time series of the 1-gram virus in French shows strong relative retweeting following global news about the early spread of COVID-19 in 2020–2021. (**F**) We observe mild spikes at the beginning of the German dialog around the withdrawal of the United Kingdom from the European Union shifting to an even balance of the 1-gram Brexit across organic and retweeted content.

In Fig. 3, we present a set of six “contagiograms.” With these expanded time series visualizations, we convey the degree to which an *n*-gram is retweeted both overall and relative to the background level of retweeting for a given language. We show both rates because retweet rates change strongly over time and variably so across languages (*34*).

Each contagiogram has three panels. The main panel at the bottom charts, as before, the rank time series for a given *n*-gram. For contagiograms running over a decade, we show rank time series in this main panel with month-scale smoothing (black line) and add a background shading in gray indicating the highest and lowest rank of each week.

The top two panels of each contagiogram capture the raw and relative social amplification for each *n*-gram. First, the top panel displays the raw RT/OT balance, the monthly relative volumes of each *n*-gram in retweets (RT, orange) and organic tweets (OT, blue)

$$R_{\tau ,t,\ell }=f_{\tau ,t,\ell }^{\left(\text{RT}\right)}/(f_{\tau ,t,\ell }^{\left(\text{RT}\right)}+f_{\tau ,t,\ell }^{\left(\text{OT}\right)})$$ (1)

When the balance of appearances in retweets outweighs those in organic tweets, *R*_τ, *t*, 𝓁_ > 0.5, we view the *n*-gram as nominally being amplified, and we add a solid background for emphasis. Second, in the middle panel of each contagiogram, we display a heatmap of the values of the relative amplification rate for *n*-gram τ in language 𝓁, $R_{\tau ,t,\ell }^{\text{rel}}$. Building on from the RT/OT balance, we define $R_{\tau ,t,\ell }^{\text{rel}}$ as

$$R_{\tau ,t,\ell }^{\text{rel}}=\frac{f_{\tau ,t,\ell }^{\left(\text{RT}\right)}/(f_{\tau ,t,\ell }^{\left(\text{RT}\right)}+f_{\tau ,t,\ell }^{\left(\text{OT}\right)})}{\sum_{\tau^{\prime }}\;f_{\tau^{\prime },t,\ell }^{\left(\text{RT}\right)}/\sum_{\tau^{\prime }}(f_{\tau^{\prime },t,\ell }^{\left(\text{RT}\right)}+f_{\tau^{\prime },t,\ell }^{\left(\text{OT}\right)})}$$ (2)

where the denominator gives the overall fraction of *n*-grams that are found in retweets on day *t* for language 𝓁. While still averaging at month scales, we now do so based on day of the week. Shades of red indicate that the relative volume of *n*-gram τ is being socially amplified over the baseline of retweets in language 𝓁, $R_{\tau ,t,\ell }^{\text{rel}}>1$, while gray encodes the opposite, $R_{\tau ,t,\ell }^{\text{rel}}<1$.

The contagiogram in Fig. 3A for the word for “kevät,” “spring” in Finnish, shows an expected annual periodicity. The word has a general tendency to appear in organic tweets more than retweets, but this is true of Finnish words in general, and we see that from the middle panel that kevät is relatively, if patchily, amplified when compared to all Finnish words. For the anticipatory periodic time series in Fig. 3B, we track references to the “Carnival of Madeira” festival, held 40 days before Easter in Brazil. We see that “Carnival” has become increasingly amplified over time and has been relatively more amplified than Portuguese words except for 2015 and 2016.

By etymological definition, renowned individuals should feature strongly in retweets (“renown” derives from “to name again”). Lionel Messi has been one of the most talked about sportspeople on Twitter over the past decade, and Fig. 3C shows that his 2-gram is strongly retweeted, by both raw and relative measures. (See also fig. S4F for the K-pop band BTS’s extreme levels of social amplification.)

Some *n*-grams exhibit a consistent weekly ampflification signal. For example, “#TGIF” is organically tweeted on Thursdays and Fridays but retweeted more often throughout the rest of the week (Fig. 3D). At least for those 2 days, individuals expressing relief for the coming weekend overwhelm any advertising from the eponymous restaurant chain.

Routinely, *n*-grams will take off in usage and amplification because of global events. In Fig. 3E, we see “virus” in French tweets holding a stable rank throughout the 2010s before jumping in response to the COVID-19 pandemic and showing mildly increased amplification levels. The word Brexit in German has been prevalent from 2016 on, balanced in terms of organic tweet and retweet appearances, and generally more spread than German 1-grams.

The contagiograms in Fig. 3 give just a sample of the rich variety of social amplification patterns that appear on Twitter. We include some further examples in figs. S4 and S5. We provide a Python package for generating arbitrary contagiograms along with further examples at https://gitlab.com/compstorylab/contagiograms. The figure-making scripts interact directly with the Storywrangler database and offer a range of configurations.

### Narratively trending storylines

Besides curating daily Zipf distributions, Storywrangler serves as an analytical tool to examine and explore the lexicon of emerging storylines in real time. Using rank-turbulence divergence (RTD) (*35*), we examine the daily rate of usage of each *n*-gram, assessing the subset of *n*-grams that have become most inflated in relative usage. For each day *t*, we compute RTD for each *n*-gram τ relative to the year before *t*′, setting the parameter α to ^1^/_4_ to examine the lexical turbulence of social media data such that

$$\delta D_{\tau }^{R}={|\frac{1}{r_{\tau ,t,\ell }^{\alpha }}-\frac{1}{r_{\tau ,t\prime ,\ell }^{\alpha }}|}^{1/(\alpha +1)};(\alpha =1/4)$$ (3)

**Fig. 4:**
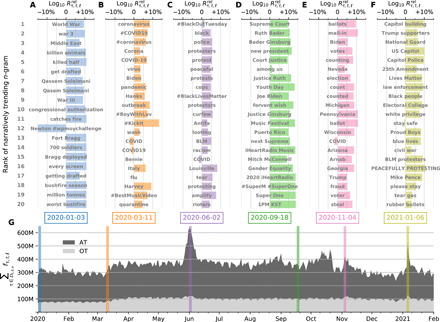
Narratively trending *n*-grams. We use RTD (*35*) to find the most narratively trending *n*-grams of each day relative to the year before in English tweets. For each day, we display the top 20 *n*-grams sorted by their RTD value on that day. We also display the relative social amplification ratio $R_{\tau ,t,\ell }^{\text{rel}}$ for each *n*-gram on a logarithmic scale, whereby positive values indicate strong social amplification of that *n*-gram via retweets and negative values imply that the given *n*-gram is often shared in originally authored tweets. (**A**) The assassination of Iranian general Qasem Soleimani by a U.S. drone strike on 3 January 2020 (blue). (**B**) World Health Organization declares COVID-19 a global pandemic on 11 March 2020 (orange). (**C**) Mass protests against racism and police brutality on 2 June 2020 (purple). (**D**) Death of U.S. Supreme Court justice Ruth Ginsburg from complications of pancreatic cancer on 18 September 2020 (green). (**E**) The 2020 U.S. presidential election held on 04 November 2020 (pink). (**F**) The deadly insurrection of the U.S. Capitol on 6 January 2021 (yellow). (**G**) Daily *n*-gram volume (i.e., number of words) for all tweets (AT, gray) and organic tweets (OT, light gray).

Although our tool uses RTD to determine marked shifts in relative usage of *n*-grams, other divergence metrics will yield similar lists. In Fig. 4, we show an example analysis of all English tweets for a few days of interest in 2020. First, we determine the top 20 narratively dominate *n*-grams of each day using RTD, leaving aside links, emojis, handles, and stop words but keeping hashtags. Second, we compute the relative social amplification ratio $R_{\tau ,t,\ell }^{\text{rel}}$ to examine whether a given *n*-gram τ is prevalent in originally authored tweets or socially amplified via retweets on day *t*. For ease of plotting, we have further chosen to display $R_{\tau ,t,\ell }^{\text{rel}}$ at a logarithmic scale. Positive values of ${\text{log}}_{10}R_{\tau ,t,\ell }^{\text{rel}}$ imply strong social amplification of τ, whereas negative values show that τ is relatively more predominant in organic tweets.

Figure 4A gives us a sense of the growing discussions and fears of a global warfare following the assassination of Iranian general Qasem Soleimani by a U.S. drone airstrike on 3 January 2020. While most of the terms are socially amplified, we note that the bigram “Newton #wpmoychallenge” was trending in organic tweets, reflecting the ongoing campaign and nomination of Cam Newton for Walter Payton NFL Man of the Year Award, an annual reward that is granted for an NFL player for their excellence and contributions.

In Fig. 4B, we see how conversations of the Coronavirus disease becomes the most prevailing headline on Twitter with the World Health Organization declaring COVID-19 a global pandemic on 11 March 2020.

In light of the social unrest sparked by the murder of George Floyd in Minneapolis, we observe the growing rhetoric of the Black Lives Matter movement on Twitter driven by an enormous increase of retweets in Fig. 4C. The top narratively trending unigram is “#BlackOutTuesday,” a newborn movement that matured overnight on social media, leading to major music platforms such as Apple and Spotify to shut down their operations on 2 June 2020 in support of the nationwide protests against racism and police brutality.

In Fig. 4D, we see the name of the U.S. Supreme Court justice Ruth Bader Ginsburg amplified on Twitter, mourning her death from complications of pancreatic cancer on 18 September 2020. We also see names of politicians embodying the heated discourse on Twitter preceding the first U.S. presidential debate. Emerging pop culture trends can also be observed in the anticipation of the first album by a K-pop South Korean band “SuperM,” entitled “Super One.”

In Fig. 4E, we see names of swing states and political candidates come to the fore during the U.S. presidential election held on 4 November 2020. We observe another surge of retweets during the storming of the U.S. Capitol by Trump supporters on 6 January 2021. Figure 4F shows the top 20 prevalent bigrams emerging on Twitter in response to the deadly insurrection.

In Fig. 4G, we display the daily *n*-gram volume (i.e., number of words) throughout the year for all tweets (AT, gray) and organic tweets (OT, light gray). We provide more examples in Appendix B and figs. S8 and S9, demonstrating the wide variety of sociocultural and sociotechnical phenomena that can be identified and examined using Storywrangler.

### Case studies

As a demonstration of our dataset’s potential value to a diverse set of disciplines, we briefly present three case studies. We analyze (i) the dynamic behavior of famous individuals’ full names and their association with the individuals’ ages, (ii) the relationship between movie revenue and anticipatory dynamics in title popularity, and (iii) the potential of social unrest–related words to predict future geopolitical risk.

**Fig. 5:**
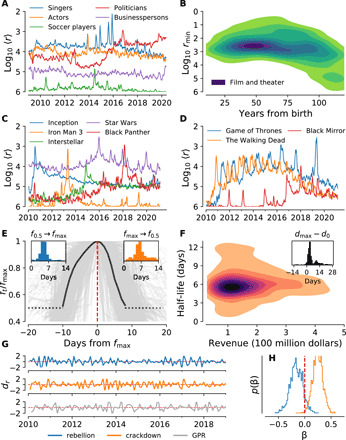
Three case studies joining Storywrangler with other data sources. (**A**) Monthly rolling average of rank 〈*r*〉 for the top five ranked Americans born in the past century in each category for a total of 960 individuals found in the Pantheon dataset (*36*). (**B**) Kernel density estimation for the top rank *r*_min_ achieved by 751 personalities in the film and theater industry as a function of their age. (**C**) Rank time series for example movie titles showing anticipation and decay. (**D**) Contrasting with (C), rank time series for TV series titles. (**E** and **F**) Time series and half-life revenue comparison for 636 movie titles with gross revenue at or above the 95th percentile released between 1 January 2010 and 31 July 2017 (*37*). (**G** and **H**) The Storywrangler dataset can also be used to potentially predict political and financial turmoil. Percent change in the words rebellion and crackdown in month *m* is significantly associated with percent change in a geopolitical risk (GPR) index in month *m* + 1 (*38*). (G) Percent change time series. (H) Distributions of coefficients of a fit linear model. See Appendices C to E for details of each study.

We examine the dialog around celebrities by cross-referencing our English 2-grams corpus with names of famous personalities from the Pantheon dataset (*36*). We searched through our English *n*-grams dataset and selected names that were found in the top million ranked 2-grams for at least 1 day between 1 January 2010 and 1 June 2020. In Fig. 5A, we display a monthly rolling average (centered) of the average rank for the top five individuals for each category 〈*r*_min(5)_〉 (see also fig. S10). In Fig. 5B, we display a kernel density estimation of the top rank achieved by any of these individuals in each industry as a function of the number of years since the recorded year of birth. We note a high density of individuals marking their best rankings between 40 and 60 years of age in the film and theater industry. Different dynamics can be observed in fig. S10 for other industries.

We next investigate the conversation surrounding major film releases by tracking *n*-grams that appear in titles for 636 movies with gross revenue above the 95th percentile during the period ranging from 1 January 2010 to 1 July 2017 (*37*). We find a median value of 3 days after release for peak normalized frequency of usage for movie *n*-grams (Fig. 5F, inset). Growth of *n*-gram usage from 50% (*f*_0.5_) to maximum normalized frequency (*f*_max_) has a median value of 5 days across our titles. The median value of time to return to *f*_0.5_ from *f*_max_ is 6 days. Looking at Fig. 5E, we see that the median shape of the spike around movie release dates tends to entail a gradual increase to peak usage and a relatively more sudden decrease when returning to *f*_0.5_. There is also slightly more spread in the time to return to *f*_0.5_ compared with the time to increase from *f*_0.5_ to *f*_max_ (Fig. 5E, insets).

In Fig. 5 (G and H), we show that changes in word usage can be associated to future changes in geopolitical risk, which we define here as “a decline in real activity, lower stock returns, and movements in capital flows away from emerging economies,” following the U.S. Federal Reserve (*38*). We chose a set of words that we a priori believed might be relevant to geopolitical risk as design variables and a geopolitical index created by the U.S. Federal Reserve as the response. We fit a linear model using the values of the predictors at month *m* to predict the value of the geopolitical risk index at month *m* + 1. Two of the words, “rebellion” and “crackdown,” have statistically significant association with changes in the geopolitical risk index (see Appendix E).

Although global events and breaking news are often recorded across conventional and modern social media platforms, Storywrangler uniquely tracks ephemeral day-to-day conversations and sociocultural trends. In creating Storywrangler, we sought to develop and maintain a large-scale daily record of everyday dialogs that is complementary to existing data sources but equally vital to identify and study emerging sociotechnical phenomena. For details about our methodology and further results, see Appendices C to E.

## DISCUSSION

With this initial effort, we aim to introduce Storywrangler as a platform enabling research in computational social science, data journalism, natural language processing, and the digital humanities. Along with phrases associated with important events, Storywrangler encodes casual daily conversation in a format unavailable through newspaper articles and books. While its utility is clear, there are many potential improvements to introduce to Storywrangler. For high volume languages, we would aim for higher temporal resolution, at the scale of minutes, and, in such an implementation, we would be limited by requiring *n*-gram counts to exceed some practical minimum. We would also want to expand the language parsing to cover continuous-script languages such as Japanese and Chinese.

Another large space of natural improvements would be to broadly categorize tweets in ways other than by language identification, while preserving privacy, such as geography, user type (e.g., people, institutions, or automated), and topic (e.g., tweets containing fake news). We note that for Twitter, features such as location and user type are more difficult to establish with as much confidence as for language identification. Increasingly by design, geographic information is limited on Twitter as are user demographics, although some aspects may be gleaned indirectly (*39*–*43*). Regardless, in this initial curation of Twitter *n*-grams, we purposefully do not attempt to incorporate any metadata beyond identified language into the *n*-gram database.

Topic-based subsets are particularly promising, as they would allow for explorations of language use, ambient framings, narratives, and conspiracy theories. Parsing into 2-grams and 3-grams makes possible certain analyses of the temporal evolution of 1-grams adjacent to an anchor 1-gram or 2-gram. Future development will enable the use of wild cards so that linguists will, in principle, be able to track patterns of popular language use in a way that the Google Books *n*-gram corpus is unable to do (*1*, *2*). Similarly, journalists and political scientists could chart *n*-grams being used around, for example, “#BlackLivesMatter” or “Trump” over time (*44*).

Looking outside of text, a major possible expansion of the instrument would be to incorporate image and video captions, a growing component of social media communications over the past decade. Moving away from Twitter, we could use Storywrangler for other platforms where social amplification is a recorded feature (e.g., Reddit, 4Chan, Weibo, and Parler).

There are substantive limitations to Twitter data, some of which are evident in many large-scale text corpora. Our *n*-gram dataset contends with popularity, allowing for the examination of story amplification, and we emphasize the importance of using contagiograms as visualization tools that go beyond presenting simple time series. Popularity, however, is notoriously difficult to measure. The main proxy that we use for popularity is the relative rate of usage of a given *n*-gram across originally authored tweets, examining how each term or phrase is socially amplified via retweets. While Twitter attempts to measure popularity by counting impressions, it is increasingly difficult to capture the number of people exposed to a tweet. Twitter’s centralized trending feature is yet another dimension that alters the popularity of terms on the platform, personalizing each user timeline and inherently amplifying algorithmic bias. We have also observed a growing passive behavior across the platform leading to an increasing preference for retweets over original tweets for most languages on Twitter during the past few years (*34*).

Twitter’s user base, while broad, is clearly not representative of the populace (*45*); is moreover compounded by the mixing of voices from people, organizations, and bots; and has evolved over time as new users have joined. Still, modern social media provides an open platform for all people to carry out conversations that matter to their lives. Storywrangler serves as instrument to depict discourse on social media at a larger scale and finer time resolution than current existing resources. Sociocultural biases that are inherently intrinsic to these platforms will be exposed using Storywrangler, which can inspire developers to enhance their platforms accordingly (*46*, *47*).

Social structures (e.g., news and social media platforms) form and reshape individual behavior, which evidently alters social structures in an algorithmic feedback loop fashion (*48*). For instance, a trending hashtag can embody a social movement (e.g., #MeToo), such that an *n*-gram may become mutually constituted to a behavioral and sociocultural revolution. Social and political campaigns can leverage an *n*-gram in their organized marketing strategies, seeking sustained collective attention on social media platforms encoded through spikes in *n*-gram usage rates. There are many examples of this emerging sociotechnical phenomenon on Twitter, ranging from civil rights (e.g., #WomensMarch) to gender identity (e.g., #LGBTQ) to political conspiracy theories (e.g., #QAnon) to academy awards promotions (e.g., #Oscar) to movie advertisement (e.g., #Avengers), etc. The Canadian awareness campaign “Bell Let’s Talk” is another example of an annual awareness campaign that subsidizes mental health institutions across Canada, donating 5 cents for every (re)tweet containing the hashtag “#BellLetsTalk” to reduce stigma surrounding mental illness. Marketing campaigns have also grasped the periodic feature of key trending *n*-grams and adjusted their language accordingly. Marketers and bots often exploit this periodicity by hijacking popular hashtags to broadcast their propaganda (e.g., including #FF and #TGIF as trending hashtags for Friday promotions).

In building Storywrangler, we have prioritized privacy by aggregating statistics to day-scale resolution for individual languages, truncating distributions, ignoring geography, and masking all metadata. We have also endeavored to make our work as transparent as possible by releasing all code associated with our framework.

Although we frame Storywrangler as a research-focused instrument akin to a microscope or telescope for the advancement of science, it does not have built-in ethical guardrails. There is potential for misinterpretation and mischaracterization of the data, whether purposeful or not. For example, we strongly caution against cherry-picking isolated time series that might suggest a particular story or social trend. Words and phrases may drift in meaning and other terms take their place. For example, “coronavirus” gave way to “covid” as the dominant term of reference on Twitter for the COVID-19 pandemic in the first 6 months of 2020 (*49*). To, in part, properly demonstrate a trend, researchers would need to at least marshal together thematically related *n*-grams and do so in a data-driven way, as we have attempted to do for our case studies. Thoughtful consideration of overall and normalized frequency of usage would also be needed to show whether a topic is changing in real volume.

In building Storywrangler, our primary goal has been to build an instrument to curate and share a rich, language-based ecology of interconnected *n*-gram time series derived from social media. We see some of the strongest potential for future work in the coupling of Storywrangler with other data streams to enable, for example, data-driven, computational versions of journalism, linguistics, history, economics, and political science.

## MATERIALS AND METHODS

### Overview of Storywrangler

We draw on a storehouse of messages comprising roughly 10% of all tweets collected from 9 September 2008 onward and covering 150+ languages. In previous work (*34*), we described how we reidentified the languages of all tweets in our collection using FastText-LID (*50*, *51*), uncovering a general increase in retweeting across Twitter over time. A uniform language reidentification was needed as Twitter’s own real time identification algorithm was introduced in late 2012 and then adjusted over time, resulting in temporal inconsistencies for long-term streaming collection of tweets (*52*). While we can occasionally observe subtle cues of regional dialects and slang, especially on a nonmainstream media platform like Twitter, we still classify them on the basis of their native languages. Date and language are the only metadata that we incorporate into our database. For user privacy in particular, we discard all other information associated with a tweet.

For each day *t* (Eastern Time encoding) and for each language 𝓁, we categorize tweets into two classes: organic tweets (OT) and retweets (RT). To quantify the relative effect of social amplification, we group originally authored posts, including the comments found in quote tweets but not the retweeted content they refer to, into what we call organic tweets. We break each tweet into 1-grams, 2-grams, and 3-grams. Although we can identify tweets written in continuous script–based languages (e.g., Japanese, Chinese, and Thai), our current implementation does not support breaking them into *n*-grams.

We accommodate all Unicode characters, including emojis, contending with punctuation as fully as possible (see Appendix A for further details). For our application, we designed a custom *n*-gram tokenizer to preserve handles, hashtags, date/time strings, and links [similar to the tweet tokenizer in the Natural Language Toolkit library (*53*)]. Although some older text tokenization toolkits followed different criteria, our protocol is consistent with modern computational linguistics for social media data (*8*, *54*).

We derive three essential measures for each *n*-gram: raw frequency (or count), normalized frequency (interpretable as probability), and rank, generating the corresponding Zipf distributions (*17*). We perform this process for all tweets (AT), organic tweets (OT), and (implicitly) retweets (RT). We then record *n*-grams along with ranks, raw frequencies, and normalized frequencies for all tweets and organic tweets in a single file, with the default ordering according to *n*-gram prevalence in all tweets.

### Notation and measures

We write an *n*-gram by τ and a day’s lexicon for language 𝓁, the set of distinct *n*-grams found in all tweets (AT) for a given date *t*, by 𝒟_*t*, 𝓁; *n*_. We write *n*-gram raw frequency as *f*_τ, *t*, 𝓁_ and compute its usage rate in all tweets written in language 𝓁 as

$$p_{\tau ,t,\ell }=\frac{f_{\tau ,t,\ell }}{\sum_{\tau \prime \in D_{t,\ell ;n}}\;f_{\tau \prime ,t,\ell }}$$ (4)

We further define the set of unique language 𝓁 *n*-grams found in organic tweets as $D_{t,\ell ;n}^{\left(\text{OT}\right)}$ and the set of unique *n*-grams found in retweets as $D_{t,\ell ;n}^{\left(\text{RT}\right)}$ (hence, $D_{t,\ell ;n}=D_{t,\ell ;n}^{\left(\text{OT}\right)}\cup D_{t,\ell ;n}^{\left(\text{RT}\right)}$). The corresponding normalized frequencies for these two subsets of *n*-grams are then

$$p_{\tau ,t,\ell }^{\left(\text{OT}\right)}=\frac{f_{\tau ,t,\ell }^{\left(\text{OT}\right)}}{\sum_{\tau \prime \in D_{t,\ell ;n}^{\left(\text{OT}\right)}}\;f_{\tau \prime ,t,\ell }^{\left(\text{OT}\right)}}\;\text{and}$$ (5)

$$p_{\tau ,t,\ell }^{\left(\text{RT}\right)}=\frac{f_{\tau ,t,\ell }^{\left(\text{RT}\right)}}{\sum_{\tau \prime \in D_{t,\ell ;n}^{\left(\text{RT}\right)}}f_{\tau \prime ,t,\ell }^{\left(\text{RT}\right)}}$$ (6)

We rank *n*-grams by raw frequency of usage using fractional ranks for ties. The corresponding notation is

$$r_{\tau ,t,\ell },r_{\tau ,t,\ell }^{\left(\text{OT}\right)},\text{and}\;r_{\tau ,t,\ell }^{\left(\text{RT}\right)}$$ (7)

### User interface

**Fig. 6:**
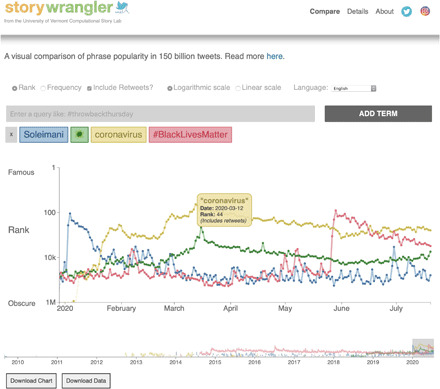
Interactive online viewer. Screenshot of the Storywrangler site showing example Twitter *n*-gram time series for the first half of 2020. The series reflect three global events: the assassination of Iranian general Qasem Soleimani by the United States on 3 January 2020, the COVID-19 pandemic (the virus emoji and coronavirus), and the Black Lives Matter protests following the murder of George Floyd by Minneapolis police (#BlackLivesMatter). The *n*-gram Storywrangler dataset for Twitter records the full ecology of text elements, including punctuation, hashtags, handles, and emojis. The default view is for *n*-gram (Zipfian) rank at the day scale (Eastern Time), a logarithmic *y* axis, and for retweets to be included. These settings can be respectively switched to normalized frequency, linear scale, and organic tweets (OT) only. The displayed time range can be adjusted with the selector at the bottom, and all data are downloadable.

We make interactive time series based on our *n*-gram dataset viewable at storywrangling.org. In Fig. 6, we show a screenshot of the site displaying rank time series for the first half of 2020 for “Soleimani,” the virus emoji, coronavirus, and #BlackLivesMatter. Ranks and normalized frequencies for *n*-grams are relative to *n*-grams with the same *n*, and in the online version, we show time series on separate axes below the main comparison plot.

For each time series, hovering over any data point will pop up an information box. Clicking on a data point will take the user to Twitter’s search results for the *n*-gram for the span of 3 days centered on the given date. All time series are shareable and downloadable through the site, as are daily Zipf distributions for the top million ranked *n*-grams in each language. Retweets may be included (the default) or excluded, and the language, vertical scale, and time frame may all be selected.

## SUPPLEMENTARY MATERIALS

Supplementary material for this article is available at http://advances.sciencemag.org/cgi/content/full/7/29/eabe6534/DC1

## References

[1] J.-B. Michel, Y. K. Shen, A. P. Aiden, A. Veres, M. K. Gray; The Google Books Team, J. P. Pickett, D. Hoiberg, D. Clancy, P. Norvig, J. Orwant, S. Pinker, M. A. Nowak, E. L. Aiden, Quantitative analysis of culture using millions of digitized books. Sci. Mag. 331, 176 (2011). 10.1126/science.1199644 PMC3279742 21163965

[2] E. A. Pechenick, C. M. Danforth, P. S. Dodds, Characterizing the Google Books corpus: Strong limits to inferences of socio-cultural and linguistic evolution. PLOS ONE 10, e0137041 (2015). 26445406 10.1371/journal.pone.0137041 PMC4596490

[3] H. Christenson, HathiTrust: A research library at web scale. Libr. Resour. Tech. Serv. 55, 93–102 (2011).

[4] M. Gerlach, F. Font-Clos, A standardized Project Gutenberg corpus for statistical analysis of natural language and quantitative linguistics. Entropy 22, 126 (2020). 10.3390/e22010126 PMC7516435 33285901

[5] E. Sandhaus, *The New York Times Annotated Corpus* (Linguistic Data Consortium, 2008).

[6] D. Beeferman, W. Brannon, D. Roy, in *Proceedings of Interspeech 2019* (International Speech Communication Association, 2019), pp. 564–568.

[7] L. Hollink, A. Bedjeti, M. van Harmelen, D. Elliott, in *Proceedings of the Tenth International Conference on Language Resources and Evaluation (LREC’16)* (European Language Resources Association, 2016), pp. 1377–1382.

[8] J. Hong, W. Crichton, H. Zhang, D. Y. Fu, J. Ritchie, J. Barenholtz, B. Hannel, X. Yao, M. Murray, G. Moriba, M. Agrawala, K. Fatahalian, Analyzing who and what appears in a decade of US cable TV news. arXiv:2008.06007 [cs.CY] (2020).

[9] W. Mieder, *Proverbs: A Handbook* (Greenwood Folklore Handbooks, Greenwood Press, 2004).

[10] J. Abello, P. Broadwell, T. R. Tangherlini, Computational folkloristics. Commun. ACM 55, 60–70 (2012).

[11] T. R. Tangherlini, P. Leonard, Trawling in the sea of the great unread: Sub-corpus topic modeling and humanities research. Poetics 41, 725–749 (2013).

[12] Q.-H. Vuong, T.-M. Ho, H.-K. Nguyen, T.-T. Vuong, Healthcare consumers’ sensitivity to costs: A reflection on behavioural economics from an emerging market. Palgrave Commun. 4, 70 (2018).

[13] J. T. Woolley, G. Peters, The American Presidency Project (1999); www.presidency.ucsb.edu/.

[14] R. B. Primack, H. Higuchi, A. J. Miller-Rushing, The conservation and management of biodiversity in Japan. Biol. Conserv. 142, 1881–1964 (2009).

[15] J. Allen, B. Howland, M. Mobius, D. Rothschild, D. J. Watts, Evaluating the fake news problem at the scale of the information ecosystem. Sci. Adv. 6, eaay3539 (2020). 32284969 10.1126/sciadv.aay3539 PMC7124954

[16] C. S. Sanders Peirce, Prolegomena to an apology for pragmaticism. Monist 16, 492–546 (2015).

[17] G. K. Zipf, *Human Behaviour and the Principle of Least-Effort* (Addison-Wesley, 1949).

[18] J. Bohannon, Google opens books to new cultural studies. Science 330, 1600 (2010). 21163974 10.1126/science.330.6011.1600

[19] A. Koplenig, The impact of lacking metadata for the measurement of cultural and linguistic change using the Google Ngram data sets—Reconstructing the composition of the German corpus in times of WWII. Digit. Scholarsh. Humanit. 32, 169–188 (2015).

[20] E. A. Pechenick, C. M. Danforth, P. S. Dodds, Is language evolution grinding to a halt? The scaling of lexical turbulence in English fiction suggests it is not. J. Comput. Sci. 21, 24–37 (2017).

[21] J. Merritt, S. Niequist, *Learning to Speak God from Scratch: Why Sacred Words Are Vanishing—And How We Can Revive Them* (Crown Publishing Group, 2018).

[22] S. Hong, D. Nadler, in *Proceedings of the 12th Annual International Digital Government Research Conference: Digital Government Innovation in Challenging Times, dg.o ‘11* (Association for Computing Machinery, 2011), pp. 182–186.

[23] A. Younus, M. A. Qureshi, F. F. Asar, M. Azam, M. Saeed, N. Touheed, in *2011 International Conference on Advances in Social Networks Analysis and Mining* (IEEE, 2011), pp. 618–623.

[24] T. Sakaki, M. Okazaki, Y. Matsuo, in *Proceedings of the 19th International Conference on World Wide Web, WWW ‘10* (Association for Computing Machinery, 2010), pp. 851–860.

[25] G. Pickard, W. Pan, I. Rahwan, M. Cebrian, R. Crane, A. Madan, A. Pentland, Time-critical social mobilization. Science 334, 509–512 (2011). 22034432 10.1126/science.1205869

[26] H. Gao, G. Barbier, R. Goolsby, Harnessing the crowdsourcing power of social media for disaster relief. IEEE Intell. Syst. 26, 10–14 (2011).

[27] V. Lampos, N. Cristianini, in *2010 2nd International Workshop on Cognitive Information Processing* (IEEE, 2010), pp. 411–416.

[28] A. Culotta, in *Proceedings of the First Workshop on Social Media Analytics, SOMA 10* (Association for Computing Machinery, 2010), pp. 115–122.

[29] Z. C. Steinert-Threlkeld, D. Mocanu, A. Vespignani, J. Fowler, Online social networks and offline protest. EPJ Data Sci. 4, 19 (2015).

[30] D. R. Dewhurst, T. Alshaabi, D. Kiley, M. V. Arnold, J. R. Minot, C. M. Danforth, P. S. Dodds, The shocklet transform: A decomposition method for the identification of local, mechanism-driven dynamics in sociotechnical time series. EPJ Data Sci. 9, 3 (2020).

[31] P. S. Dodds, J. R. Minot, M. V. Arnold, T. Alshaabi, J. L. Adams, D. R. Dewhurst, A. J. Reagan, C. M. Danforth, Fame and ultrafame: Measuring and comparing daily levels of ‘being talked about’ for United States’ presidents, their rivals, God, countries, and K-pop. arXiv:1910.00149 [physics.soc-ph] (2019).

[32] H. Choi, H. Varian, Predicting the present with Google Trends. Econ. Record 88, 2–9 (2012).

[33] J. K. Armstrong, How Sherlock Holmes changed the world (2016); www.bbc.com/culture/article/20160106-how-sherlock-holmes-changed-the-world.

[34] T. Alshaabi, D. R. Dewhurst, J. R. Minot, M. V. Arnold, J. L. Adams, C. M. Danforth, P. S. Dodds, The growing amplification of social media: Measuring temporal and social contagion dynamics for over 150 languages on Twitter for 2009–2020. EPJ Data Sci. 10, 15 (2021). 33816048 10.1140/epjds/s13688-021-00271-0 PMC8010293

[35] P. S. Dodds, J. R. Minot, M. V. Arnold, T. Alshaabi, J. L. Adams, D. R. Dewhurst, T. J. Gray, M. R. Frank, A. J. Reagan, C. M. Danforth, Allotaxonometry and rank-turbulence divergence: A universal instrument for comparing complex systems. arXiv:2002.09770 [physics.soc-ph] (2020).

[36] A. Z. Yu, S. Ronen, K. Hu, T. Lu, C. A. Hidalgo, Pantheon 1.0, a manually verified dataset of globally famous biographies. Sci. Data 3, 1–16 (2016). 10.1038/sdata.2015.75 PMC4700860 26731133

[37] F. M. Harper, J. A. Konstan, The movielens datasets: History and context. ACM Trans. Inter. Intell. Syst. 5, 1–19 (2015).

[38] D. Caldara, M. Iacoviello, *FRB International Finance Discussion Paper* (Federal Reserve System, 2018).

[39] W. Liu, D. Ruths, *AAAI Spring Symposium: Analyzing Microtext* (AAAI, 2013), vol. SS-13-01 of *AAAI Technical Report*.

[40] R. Cohen, D. Ruths, *Proceedings of the International AAAI Conference on Web and Social Media* (AAAI, 2013), vol. 7.

[41] D. Preoţiuc-Pietro, S. Volkova, V. Lampos, Y. Bachrach, N. Aletras, Studying user income through language, behaviour and affect in social media. PLOS ONE 10, e0138717 (2015). 26394145 10.1371/journal.pone.0138717 PMC4578862

[42] M. Malik, H. Lamba, C. Nakos, J. Pfeffer, *Proceedings of the International AAAI Conference on Web and Social Media* (AAAI, 2015), vol. 9.

[43] X. Zheng, J. Han, A. Sun, A survey of location prediction on Twitter. IEEE Trans. Knowl. Data Eng. 30, 1652–1671 (2018).

[44] P. S. Dodds, J. R. Minot, M. V. Arnold, T. Alshaabi, J. L. Adams, A. J. Reagan, C. M. Danforth, Computational timeline reconstruction of the stories surrounding Trump: Story turbulence, narrative control, and collective chronopathy. arXiv:2008.07301 [physics.soc-ph] (2020). 10.1371/journal.pone.0260592 PMC8654215 34879105

[45] J. Mellon, C. Prosser, Twitter and Facebook are not representative of the general population: Political attitudes and demographics of British social media users. Res. Polit. 4, 2053168017720008 (2017).

[46] S. L. Blodgett, L. Green, B. O’Connor, in *Proceedings of the 2016 Conference on Empirical Methods in Natural Language Processing* (Association for Computational Linguistics, 2016), pp. 1119–1130.

[47] A. Koenecke, A. Nam, E. Lake, J. Nudell, M. Quartey, Z. Mengesha, C. Toups, J. R. Rickford, D. Jurafsky, S. Goel, Racial disparities in automated speech recognition. Proc. Natl. Acad. Sci. U.S.A. 117, 7684–7689 (2020). 32205437 10.1073/pnas.1915768117 PMC7149386

[48] A. Giddens, *The Constitution of Society: Outline of the Theory of Structuration* (University of California Press, 1984).

[49] T. Alshaabi, M. V. Arnold, J. R. Minot, J. L. Adams, D. R. Dewhurst, A. J. Reagan, R. Muhamad, C. M. Danforth, P. S. Dodds, How the world’s collective attention is being paid to a pandemic: COVID-19 related n-gram time series for 24 languages on Twitter. PLOS ONE 16, e0244476 (2021). 33406101 10.1371/journal.pone.0244476 PMC7787459

[50] A. Joulin, E. Grave, P. Bojanowski, T. Mikolov, in *Proceedings of the 15th Conference of the European Chapter of the Association for Computational Linguistics: Volume 2, Short Papers* (Association for Computational Linguistics, 2017), pp. 427–431.

[51] P. Bojanowski, E. Grave, A. Joulin, T. Mikolov, Enriching word vectors with subword information. Trans. Assoc. Comput. Linguist. 5, 135–146 (2017).

[52] P. S. Dodds, J. R. Minot, M. V. Arnold, T. Alshaabi, J. L. Adams, D. R. Dewhurst, A. J. Reagan, C. M. Danforth, Long-term word frequency dynamics derived from Twitter are corrupted: A bespoke approach to detecting and removing pathologies in ensembles of time series. arXiv:2008.11305 [physics.soc-ph] (2020).

[53] E. Loper, S. Bird, in *Proceedings of the ACL-02 Workshop on Effective Tools and Methodologies for Teaching Natural Language Processing and Computational Linguistics—Volume 1*, *ETMTNLP ‘02* (Association for Computational Linguistics, 2002), pp. 63–70.

[54] E. Bevensee, M. Aliapoulios, Q. Dougherty, J. Baumgartner, D. McCoy, J. Blackburn, SMAT: The social media analysis toolkit, in *Proceedings of the 14th International AAAI Conference on Web and Social Media* (2020), vol. 14.

[55] Q. Ke, Y.-Y. Ahn, C. R. Sugimoto, A systematic identification and analysis of scientists on Twitter. PLOS ONE 12, e0175368 (2017). 28399145 10.1371/journal.pone.0175368 PMC5388341

[56] H. A. Simon, On a class of skew distribution functions. Biometrika 42, 425–440 (1955).

[57] D. D. S. Price, A general theory of bibliometric and other cumulative advantage processes. J. Am. Soc. Inf. Sci. 27, 292–306 (1976).

[58] B. M. Hill, A simple general approach to inference about the tail of a distribution. Ann. Statist. 3, 1163–1174 (1975).

[59] D. M. W. Powers, *New Methods in Language Processing and Computational Natural Language Learning* (Association for Computational Linguistics, 1998).

[60] S. T. Piantadosi, Zipf’s word frequency law in natural language: A critical review and future directions. Psychon. Bull. Rev. 21, 1112–1130 (2014). 24664880 10.3758/s13423-014-0585-6 PMC4176592

[61] E. Bokányi, D. Kondor, G. Vattay, Scaling in words on Twitter. R. Soc. Open Sci. 6, 190027 (2019). 31824682 10.1098/rsos.190027 PMC6837183

[62] J. Pfeffer, K. Mayer, F. Morstatter, Tampering with Twitter’s sample API. EPJ Data Sci. 7, 50 (2018).

[63] J. R. Williams, P. R. Lessard, S. Desu, E. M. Clark, J. P. Bagrow, C. M. Danforth, P. S. Dodds, Zipf’s law holds for phrases, not words. Nat. Sci. Rep. 5, 12209 (2015). 10.1038/srep12209 PMC4531284 26259699

[64] M. D. Hoffman, A. Gelman, The No-U-Turn sampler: Adaptively setting path lengths in Hamiltonian monte carlo. J. Mach. Learn. Res. 15, 1593–1623 (2014).

[65] A. Gelman, D. B. Rubin, Inference from iterative simulation using multiple sequences. Statist. Sci. 7, 457–472 (1992).

[66] E. Chenoweth, M. J. Stephan, Drop your weapons: When and why civil resistance works. Foreign Aff. 93, 94 (2014).

